# Manual Dexterity Shows Greater Discretionary Value than Sensor-Based Gait and Balance Measures in Identifying Early Functional Impairment in Multiple Sclerosis

**DOI:** 10.3390/s26061866

**Published:** 2026-03-16

**Authors:** Mousa Hujirat, Alon Kalron

**Affiliations:** 1 School of Public Health, Gray Faculty of Medical and Health Sciences, Tel-Aviv University, Tel-Aviv 6139001, Israel; mousahujirat@mail.tau.ac.il; 2 Department of Physical Therapy, School of Health Professions, Gray Faculty of Medical and Health Sciences, Tel-Aviv University, Tel-Aviv 6139001, Israel; 3 Multiple Sclerosis Center, Sheba Medical Center, Tel Hashomer, Ramat-Gan 5262100, Israel; 4 Sagol School of Neuroscience, Tel-Aviv University, Tel-Aviv 6139001, Israel

**Keywords:** multiple sclerosis, sensor-based, gait, balance, upper limb, manual dexterity

## Abstract

**Objective**: To determine which physical clinical test best differentiates minimally impaired people with MS (pwMS) from healthy controls and to compare the discriminatory value of upper limb clinical assessments with sensor-based gait and postural control measures. **Methods**: Forty-one participants (21 pwMS, 20 matched healthy controls) completed a single testing session including upper limb clinical assessments (Nine-Hole Peg Test [9HPT], grip strength), gait (Timed 25-Foot Walk, Six-Minute Walk Test, and cognitive–walking dual task), and static balance assessments using wearable inertial sensors (APDM Mobility Lab system). Dual-task costs (DTCs) were calculated for gait parameters. Between-group comparisons were performed using independent *t*-tests. Pearson correlation analyses were conducted to examine interrelationships among gait variables, and a parsimonious binary logistic regression model was constructed, including non-dominant 9HPT and dual-task walking speed. Receiver operating characteristic (ROC) analyses were performed to evaluate discriminative performance and determine the optimal 9HPT cutoff. **Results**: PwMS performed significantly slower on the 9HPT for both hands (*p* ≤ 0.006) and demonstrated reduced walking performance and higher gait DTCs (*p* ≤ 0.041) compared with controls. No significant group differences were observed in grip strength or sensor-based postural control. In multivariable analysis, the overall model was significant (*p* < 0.001; Nagelkerke R^2^ = 0.49), and the non-dominant 9HPT remained the only independent predictor of group status (OR = 1.75, 95% CI [1.17–2.61]), whereas dual-task walking speed was not significant after adjustment. ROC analysis demonstrated good discriminative ability for the non-dominant 9HPT (AUC = 0.84, 95% CI [0.71–0.97]) and acceptable discrimination for dual-task walking speed (AUC = 0.75, 95% CI [0.60–0.90]). The optimal 9HPT cutoff was ≥21.4 s, yielding 71% sensitivity and 100% specificity in this cohort. **Conclusions**: Manual dexterity of the non-dominant hand may serve as a sensitive screening marker of early functional impairment in MS, demonstrating greater discriminatory value than sensor-based gait and balance measures. These findings support the inclusion of upper limb dexterity testing in the routine assessment of minimally impaired pwMS. Validation in larger, longitudinal cohorts is warranted.

## 1. Introduction

Multiple sclerosis (MS) is a chronic, immune-mediated disorder of the central nervous system characterized by inflammation, demyelination, and neurodegeneration [1]. It typically manifests between ages 20 and 40, with a female-to-male ratio of approximately 3:1 [2]. These pathological processes lead to motor, sensory, and cognitive impairments that contribute to disability and reduced quality of life [3]. Among these, motor dysfunction is one of the most common symptoms, affecting both upper and lower limbs as well as mobility and balance [4]. Importantly, subtle motor deficits may emerge even in the early stages of the disease, among individuals classified as minimally disabled according to the Expanded Disability Status Scale (EDSS) [5,6,7]. Detecting these early changes is crucial for timely intervention and effective disease management.

A range of clinical tests is routinely used to assess physical function in people with MS (pwMS), including the Nine-Hole Peg Test for fine motor control, the Timed 25-Foot Walk and Six-Minute Walk Test for gait and endurance, as well as static balance tests such as the Romberg test for postural control [8]. These measures were selected because they represent core functional domains commonly affected in MS and are widely incorporated into clinical and research assessment frameworks, including components of the Multiple Sclerosis Functional Composite (MSFC), which integrates upper-limb dexterity and short-distance gait speed as standardized outcome measures [9,10]. The 9HPT specifically captures upper-limb dexterity, which is known to decline even in early disease stages, while the T25FWT and 6MWT assess complementary aspects of mobility, including short-distance gait speed and endurance capacity. Static postural control tasks were included to evaluate balance stability, a frequent contributor to functional limitation and fall risk in pwMS.

However, their ability to detect subtle deficits among minimally disabled individuals remains uncertain. Moreover, most studies examining physical performance in minimally impaired pwMS have assessed each domain separately, limiting the ability to determine which domain provides the greatest discriminatory value within the same cohort.

In recent years, instrumented assessment tools, such as wearable motion sensors and computerized gait and balance systems, have gained attention as complementary approaches to clinical testing in the MS population [11]. These technologies enable preciseobjective quantification of movement, allowing detection of subtle motor alterations that are not captured by traditional timed or observational tests. Additionally, they provide detailed insight into movement dynamics and compensatory mechanisms commonly observed in pwMS. The inclusion of sensor-based measures in the present study was therefore intended to determine whether quantitative spatiotemporal parameters provide incremental discriminatory value beyond standard clinical tests in minimally impaired individuals.

The present study had several aims. First, to identify which physical measures best differentiate minimally impaired pwMS from healthy controls. Second, to determine whether these differences are more prominent in the upper or lower extremities. Third, to examine whether sensor-based gait and balance measures provide an advantage over standard clinical assessments of upper limb function in distinguishing between the two groups. A clearer understanding of these relationships may assist clinicians in selecting the most sensitive clinical and sensor-based assessment tools and support the early implementation of targeted rehabilitation interventions to preserve physical function in pwMS.

## 2. Methods

### 2.1. Study Design and Participants

After obtaining local ethical approval, a convenience sample of 41 participants, including 21 pwMS (16 women, aged 33.3 (SD = 9.9)) from the Multiple Sclerosis Center, Sheba Medical Center, Tel-Hashomer, Israel, and 20 age- and gender-matched healthy controls (16 women, aged 33.0 (SD = 8.8)). Participants were recruited through direct contact with the study’s staff or local advertising. Inclusion criteria for pwMS included: (1) a diagnosis of MS according to the revised McDonald Criteria 2017 [12], (2) scores between 0 and 2.0 on the Expanded Disability Status Scale (EDSS) [13], (3) age equal or above 18, (4) disease duration under 5 years, and (5) treatment with first-line DMT. Exclusion criteria included: (1) orthopedic and/or cardiovascular disorders that could negatively affect the upper and/or lower limb function, (2) major depression or cognitive decline limiting the ability to understand the study instructions or complete the study protocol, (3) pregnancy, and (4) MS clinical relapse or treatment with corticosteroid therapy within three months prior to the examination. Healthy controls were adults without any known neurological, orthopedic, cardiovascular, or respiratory disorders that could affect upper or lower limb function, and without major psychiatric or cognitive conditions that would limit their ability to complete the study protocol. The study was approved by the Sheba Institutional Review Board (Ref# SMC-0022-22). All subjects signed an informed consent form prior to participation.

### 2.2. Study Protocol

Verification of MS diagnosis, disease duration, and EDSS scores was performed by the treating neurologist. In cases of uncertainty regarding the EDSS rating, the neurologist was contacted for clarification. All physical outcome measures were administered and recorded by an experienced physical therapist during a single session conducted at the Physical Therapy Department of the Sheba MS Center, between 8:00 and 12:00 p.m. To minimize potential order or fatigue effects, the sequence of physical tests was randomized across participants. In addition to the objective physical assessments, each participant completed three self-reported outcome measures: perceived fatigue (MFIS) [14], perceived mobility (MSWS-12) [15], and the impact of MS on daily life (MSIS-29) [16].

### 2.3. Outcome Measures

#### 2.3.1. Upper Limb

Upper limb measurements included assessments of manual dexterity and grip strength. Dexterity was evaluated using the Nine-Hole Peg Test (9HPT), a standardized measure of fine motor function widely applied in MS research and clinical practice. Participants were instructed to place one peg at a time into nine holes, then remove them as quickly as possible. The time required to complete the task with each hand was recorded, with shorter completion times indicating better manual dexterity. The 9HPT is sensitive to changes in upper extremity function and is a recommended component of the Multiple Sclerosis Outcome Assessments Consortium [17].

Grip strength was assessed using a handheld Jamar dynamometer, a reliable and valid instrument for evaluating upper limb strength in pwMS. Participants were seated with their elbows flexed at 90°, their forearms in a neutral position, and their wrists slightly extended. Each hand was tested three times, and the highest value (in kilograms) was recorded as the final score. Grip strength is a sensitive indicator of upper extremity function and has been shown to correlate with overall disability in pwMS [18].

#### 2.3.2. Gait

Gait measurements included the Timed 25-Foot Walk Test (T25FWT), the 6-Minute Walk Test (6MWT), and a Cognitive Walking Dual Task Test (Cognitive-walking DT). The T25FWT is the most common quantitative measure of mobility in MS. Participants were instructed to walk 25 feet as quickly and safely as possible, and the time to complete the distance was recorded in seconds. Two trials were performed, and the average time was used for analysis. The T25FWT is a component of the Multiple Sclerosis Functional Composite and serves as a reliable and sensitive indicator of walking speed and ambulatory function in individuals with MS [10].

The APDM Mobility Lab System (v2) (Opal sensors, APDM, Portland, OR, USA) was used to collect five key spatiotemporal gait parameters (walking speed, cadence, stride length, gait cycle, and total distance) during the 6MWT and Cognitive-walking DT. The system uses three wireless inertial sensors secured by elastic straps, positioned on the lower back and bilaterally on the dorsum of each foot. The reliability and validity of the Mobility Lab system have been previously established in pwMS [19].

The 6MWT is a standardized measure of walking endurance and functional exercise capacity. Participants were instructed to walk as far as possible along a flat, straight 25-m course for six minutes. The 6MWT has demonstrated good reliability and validity in pwMS, reflecting real-world walking ability and fatigue-related limitations [20].

For the cognitive-walking DT, each participant completed two consecutive tests, separated by a one-minute break. The first consisted of normal walking for one minute, representing the single-task condition. Subsequently, participants performed the cognitive-walking dual task, which incorporated the Modified Word List Generation (WLG) test while walking. The WLG is a neuropsychological measure commonly used to evaluate cognitive function in MS [21]. During the dual-task condition, participants were instructed to generate as many words as possible within a given category (e.g., words beginning with the Hebrew letter Bet [B]). Dual task cost (%) (DTC) was calculated for each of the five gait parameters using the following formula: [(DT − ST)/ST] × 100.

#### 2.3.3. Static Postural Control

Postural control metrics were also collected using the APDM Mobility Lab system. Each participant completed a sequence of three consecutive tests under three different task conditions, with a one-minute break between tasks. Each task was repeated three times, for 30 s per trial, followed by a 30-second rest period. The task conditions were as follows: (1) Eyes open: participants were instructed to maintain a normal standing position (9 cm gap between the medial malleoli, 7° toe-out) as steadily as possible while visually focusing on a dot positioned 1 m in front of them. (2) Eyes closed: identical to the eyes open condition, but with eyes closed. (3) Tandem: identical to the eyes open condition except for foot placement, with one foot positioned directly in front of the other such that the heel of the front foot touches the toes of the back foot. For each task condition, the following outcome measures were extracted: sway area (mm^2^/s^2^), mean velocity (m/s), and path length (mm).

### 2.4. Statistical Analysis

Descriptive statistics were used to summarize the demographic and clinical characteristics of the study groups. Between-group comparisons (healthy controls vs. pwMS) were performed using independent *t*-tests. In addition, effect sizes (Cohen’s d) were calculated for between-group comparisons using the pooled standard deviation and interpreted according to conventional thresholds (small ≈ 0.2, medium ≈ 0.5, large ≥ 0.8). The *t*-tests were conducted separately for each physical domain: upper limb function, gait, and static postural control. Pearson correlation analyses were performed to examine relationships among gait variables. Substantial intercorrelations were observed among several spatiotemporal gait parameters, reflecting mechanical interdependence (e.g., walking speed and total distance; cadence and gait cycle duration). To reduce multicollinearity and avoid model overfitting given the modest sample size, only one representative gait variable (dual-task walking speed) was included in the logistic regression model. A binary logistic regression analysis was then performed, including non-dominant 9HPT and dual-task walking speed as predictors of group allocation (MS vs. healthy controls). Receiver operating characteristic (ROC) analyses were conducted to evaluate the discriminative performance of key outcome measures. The area under the curve (AUC) with 95% confidence intervals was calculated using a nonparametric approach. The optimal cutoff for the 9HPT was determined using the Youden index. Discrimination accuracy was interpreted according to conventional criteria (AUC 0.7–0.8 acceptable, 0.8–0.9 good, >0.9 excellent). All statistical analyses were conducted using SPSS software, version 29 (IBM Corp., Armonk, NY, USA). Statistical significance was set at *p* < 0.05.

## 3. Results

Table 1 presents the demographic and clinical characteristics of the study sample. No significant differences were observed between the groups in age or sex distribution. PwMS exhibited a minimal level of disability (mean EDSS = 1.3) and a relatively recent diagnosis (mean disease duration = 2 years). Self-reported mobility limitations were mild (mean MSWS-12 = 18.2).

As shown in Table 2, pwMS performed significantly slower than healthy controls on the 9HPT for both the dominant (20.5 ± 2.8 s vs. 18.5 ± 1.9 s; *p* = 0.006) and non-dominant (21.9 ± 2.6 s vs. 19.1 ± 1.5 s; *p* < 0.001) hands, whereas no group differences were observed in grip strength. Regarding gait performance (Table 3), pwMS walked significantly slower on the T25FWT (4.6 ± 1.0 s vs. 3.9 ± 0.6 s; *p* = 0.004) and covered a shorter distance in the 6MWT (423.7 ± 6.9 m vs. 464.7 ± 44.0 m; *p* = 0.003) compared with controls. Mean stride length and walking speed were reduced among PwMS in both single-task (normal walking) and cognitive dual-task conditions (*p* ≤ 0.033). Dual-task costs (DTCs) were significantly greater in pwMS for total distance, cadence, gait cycle, and walking speed (*p* ≤ 0.041), indicating a greater performance decrement under cognitive load. Static postural control results (Table 3) revealed no significant group differences in sway area, mean velocity, or path length across any of the task conditions (eyes open, eyes closed, or tandem stance).

Given the observed intercorrelations among gait variables, a parsimonious multivariable model was constructed. In the revised logistic regression model including non-dominant 9HPT and dual-task walking speed (Table 4), the overall model was statistically significant (Omnibus χ^2^(2) = 18.79, *p* < 0.001) and explained 49% of the variance in group allocation (Nagelkerke R^2^ = 0.49). The non-dominant 9HPT remained a significant independent predictor of group status (B = 0.558, SE = 0.204, *p* = 0.006, OR = 1.75, 95% CI [1.17–2.61]). In contrast, dual-task walking speed was not independently associated with group allocation after adjustment (B = –5.73, SE = 3.19, *p* = 0.073). ROC analyses further evaluated discriminative performance. The non-dominant 9HPT demonstrated good discrimination (AUC = 0.84, 95% CI [0.71–0.97], *p* < 0.001), whereas dual-task walking speed showed acceptable but lower accuracy (AUC = 0.75, 95% CI [0.60–0.90], *p* = 0.001). The optimal 9HPT cutoff determined using the Youden index was ≥21.4 s, yielding a sensitivity of 71% and specificity of 100% for distinguishing pwMS from healthy controls.

**Figure 1:**
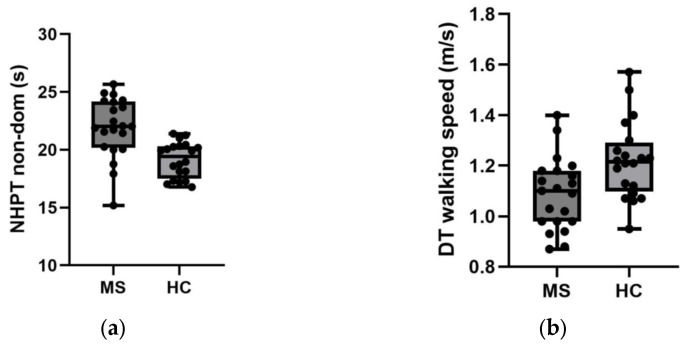
(**a**) Non-dominant nine-hole peg test scores according to study groups. (**b**) Dual-task walking speed values according to study groups.

These findings indicate that although gait performance carries a discriminative signal, upper limb dexterity provides superior classification accuracy in this minimally impaired cohort. Figure 1 present the scores of the non-dominant 9HPT and dual-task walking speed, respectively.

## 4. Discussion

The primary objective of this study was to identify the physical measure that most effectively differentiates minimally impaired pwMS from healthy adults. Although participants with MS demonstrated poorer performance on walking tests than controls, the strongest parameter distinguishing between groups was upper limb function, specifically manual dexterity of the non-dominant hand. Notably, this measure outperformed even the quantitative gait parameters obtained during both normal walking and dual-task cognitive walking. This finding remained robust after adjustment for dual-task walking speed in a multivariable regression model, in which the 9HPT retained independent significance while gait speed did not.

The present findings align with previous research, which shows that alterations in walking [5,6] and upper limb function [22,23] can occur during the very early phases of MS. However, the relatively unexpected observation that the 9HPT was the primary differentiating measure warrants further consideration. The test requires participants to repeatedly place and remove nine pegs into nine holes, one at a time, as quickly as possible. Approximately 53% of the variance in 9HPT performance has been attributed to muscle strength, tactile sensitivity of the thumb, and the presence of intention tremor [24]. Although the current study did not directly assess these factors, it is plausible that subtle alterations in sensory feedback or fine motor coordination of the non-dominant hand contributed to the observed differences. In contrast to walking, which benefits from redundant neural pathways and compensatory mechanisms, fine hand movements depend on precise integration of sensory input, including tactile feedback from the thumb, and motor output [25]. Therefore, even minor disruptions in corticospinal or cerebellar pathways, which may still permit near-normal gait, could lead to measurable slowing of manual dexterity, particularly in the less dominant hand. While this interpretation remains speculative, it may help explain why the 9HPT emerged as a more sensitive indicator of early dysfunction than other physical performance metrics.

In contrast to the walking trials, no significant differences were found between groups in the static balance tests. This may reflect the high degree of sensory redundancy underlying postural control. Balance relies on visual, vestibular, and somatosensory input, and in early MS, mild dysfunction in one system may be compensated for by the others, allowing a stable upright stance. Additionally, the tasks used (eyes open, eyes closed, tandem stance) involved bipedal standing and may have been insufficiently challenging for minimally impaired participants, potentially resulting in a ceiling effect. More demanding or dynamic paradigms may be required to detect subtle postural control deficits at this stage of the disease. Importantly, the absence of static balance differences further supports the notion that early dysfunction may preferentially affect fine motor integration rather than gross postural stability.

Interestingly, dual-task walking did not enhance group discrimination beyond single-task gait. Although dual-task paradigms are often considered sensitive to subtle neurological dysfunction, their utility may depend on disease severity and task complexity. In this cohort of minimally impaired individuals with short disease duration, cognitive-motor interference may not yet be sufficiently pronounced to produce measurable deficits. Moreover, the cognitive task employed may not have imposed enough attentional demand to challenge integration processes. Notably, ROC analysis demonstrated that dual-task walking speed did show acceptable discriminative ability; however, its classification accuracy was lower than that of the non-dominant 9HPT. These findings suggest that dual-task gait may be more informative at later stages of MS progression than at the earliest phases. Furthermore, even when dual-task walking speed was included in the multivariable model, it did not independently predict group allocation, underscoring the relative sensitivity of upper limb dexterity in this cohort.

From a physical medicine and rehabilitation perspective, these findings highlight the importance of comprehensive functional screening in the early stages of MS. While gait and balance assessments remain central components of rehabilitation evaluation, our results suggest that subtle impairments in upper limb function, particularly manual dexterity of the non-dominant hand, may emerge earlier and be more sensitive to minimal disability. Early identification of such impairments may enable clinicians to initiate targeted upper limb rehabilitation strategies before more overt mobility limitations develop. These findings should be viewed as hypothesis-generating and intended to inform clinical screening and assessment practices rather than predictive modeling. Future longitudinal studies are warranted to determine whether early deficits in manual dexterity are associated with subsequent declines in mobility, balance, or overall functional independence.

### Strengths and Limitations

This study has several strengths. It employed a comprehensive multimodal assessment, combining upper-limb, gait, and postural control evaluations within the same cohort, enabling direct comparisons across motor domains that are often studied separately. The inclusion of instrumented measures alongside standard clinical tests provided objective, quantitative data to complement traditional assessments. In addition, incorporating a cognitive–walking dual-task paradigm enhanced ecological validity and revealed subtle deficits not captured under single-task conditions. Overall, this study should be considered a pilot, hypothesis-generating investigation designed to explore early motor differences rather than to establish definitive diagnostic thresholds. The use of a parsimonious multivariable modeling strategy further strengthens the robustness of the primary finding by reducing multicollinearity among gait parameters.

However, several limitations should be noted. The sample size was relatively small, which may limit the generalizability of the findings and reduce statistical power to detect weaker effects. Moreover, although participants were matched for age and sex, other potential confounders, such as physical activity, were not fully controlled. Finally, the use of a convenience sample from a single MS center may introduce selection bias, warranting replication in larger, multicenter cohorts. Additionally, the regression model was limited to a small number of predictors to avoid overfitting, so other potentially relevant factors were not examined simultaneously. Despite these limitations, the findings underscore the potential value of upper-limb assessments, particularly manual dexterity measures, as sensitive indicators of early functional changes in pwMS.

## 5. Conclusions

Among minimally impaired individuals with multiple sclerosis, manual dexterity, particularly of the non-dominant hand, was the most sensitive clinical measure distinguishing patients from healthy controls, demonstrating superior and independent discriminatory value compared with both traditional and sensor-based gait and balance assessments. These findings support the inclusion of upper limb dexterity testing in routine clinical evaluations of early MS and suggest that reliance on mobility measures alone may overlook meaningful functional impairment detectable through clinical assessment. Although gait measures demonstrated acceptable discriminative ability, upper limb dexterity showed greater classification accuracy and remained independently predictive in multivariable analysis. Larger, longitudinal studies are needed to determine whether early changes in manual dexterity predict later disability progression and to inform the development of targeted, stage-specific rehabilitation interventions. Furthermore, the proposed 9HPT cutoff should be validated in larger cohorts before clinical implementation.

## Figures and Tables

**Table 1:** Demographic and clinical characteristics of the study sample.

Variable	MS (*n* = 21)	Healthy Controls (*n* = 20)	*p*-Value
Age	33.3 (9.9)	33.0 (8.8)	0.90
Gender (M/F)	6/15	6/14	1.00
Height (cm)	166.3 (8.9)	166.4 (10.0)	0.98
Disease duration (years)	2.0 (0.9)	-	-
EDSS (Score)	1.3 (0.7)	-	-
Perceived fatigue (MFIS)	29.6 (18.6)	-	-
Physical subscale	12.1 (8.6)	-	-
Cognitive subscale	15.6 (9.8)	-	-
Psychosocial subscale	1.9 (1.9)	-	-
Perceived mobility (MSWS-12)	18.2 (8.7)	-	-
Impact on daily life (MSWS-29)	49.8 (19.6)	-	-

Scores are presented as Mean (S.D.).

**Table 2:** Upper limb test scores according to study groups.

Variable	MS (*n* = 21)	Healthy Controls (*n* = 20)	*p*-Value	Cohen’s d Effect Size
Nine-hole peg test (s)				
*Dominant*	20.5 (2.8)	18.5 (1.9)	**0.006**	0.883
*Non-dominant*	21.9 (2.6)	19.1 (1.5)	**<0.001**	1.304
Grip strength (kg/f)				
*Dominant*	30.3 (11.6)	27.5 (10.3)	0.094	0.418
*Non-dominant*	35.0 (10.8)	32.6 (10.1)	0.059	0.499

Scores are presented as Mean (S.D.).

**Table 3:** Walking and static postural control values according to study groups.

Variable	MS (*n* = 21)	Healthy Controls (*n* = 20)	*p*-Value	Cohen’s d Effect Size
Timed 25F walk test (s)	4.59 (0.96)	3.91 (0.55)	**0.004**	0.860
Six-minute walk test				
*Total distance* (m)	423.7 (46.9)	464.7 (44.0)	**0.003**	0.901
*Cadence* (steps/min)	113.5 (6.8)	116.2 (6.0)	0.095	0.883
*Gait cycle* (s)	1.06 (0.06)	1.04 (0.05)	0.088	0.417
*Mean stride length* (m)	1.24 (0.13)	1.33 (0.11)	**0.011**	0.431
*Walking speed* (m/s)	1.18 (0.13)	1.29 (0.12)	**0.003**	0.744
Normal walking (single task)				
*Total distance* (m)	72.9 (7.2)	78.1 (9.2)	**0.026**	0.627
*Cadence* (steps/min)	115.8 (6.8)	116.8 (8.4)	0.299	0.166
*Gait cycle* (s)	1.04 (0.06)	1.03 (0.06)	0.272	0.191
*Mean stride length* (m)	1.26 (0.13)	1.33 (0.13)	**0.033**	0.589
*Walking speed* (m/s)	1.21 (0.12)	1.30 (0.15)	**0.024**	0.635
Cognitive-walking (dual task)				
*Total distance* (m)	65.2 (8.4)	73.1 (9.1)	**0.003**	0.909
*Cadence* (steps/min)	108.3 (8.2)	112.3 (5.8)	**0.040**	0.560
*Gait cycle* (s)	1.11 (0.08)	1.07 (0.06)	**0.035**	0.583
*Mean stride length* (m)	1.20 (0.12)	1.30 (0.13)	**0.008**	0.789
*Walking speed* (m/s)	1.09 (0.14)	1.22 (0.15)	**0.003**	0.910
Dual task cost (%)				
*Total distance* (m)	−10.5 (8.4)	−6.2 (6.6)	**0.037**	0.573
*Cadence* (steps/min)	−6.4 (4.7)	−3.8 (3.9)	**0.030**	0.604
*Gait cycle* (s)	7.1 (5.7)	4.3 (4.3)	**0.040**	0.561
*Mean stride length* (m)	−4.4 (5.2)	−2.5 (4.0)	0.101	0.406
*Walking speed* (m/s)	−10.4 (8.3)	−6.2 (6.6)	**0.041**	0.559
Postural control—Eyes open				
*Sway area* (mm^2^/s^2^)	2.08 (1.30)	2.85 (1.89)	0.135	0.476
*Mean velocity* (m/s)	0.18 (0.06)	0.22 (0.09)	0.106	0.517
*Path length* (mm)	7.98 (2.49)	8.74 (4.87)	0.530	0.198
Postural control—Eyes closed				
*Sway area* (mm^2^/s^2^)	4.88 (4.35)	4.44 (3.33)	0.719	0.113
*Mean velocity* (m/s)	0.23 (0.09)	0.23 (0.08)	0.760	0.096
*Path length* (mm)	11.68 (4.61)	10.32 (4.65)	0.354	0.293
Postural control—Tandem with eyes open				
*Sway area* (mm^2^/s^2^)	5.67 (5.33)	6.32 (7.24)	0.371	0.104
*Mean velocity* (m/s)	0.24 (0.10)	0.27 (0.14)	0.235	0.228
*Path length* (mm)	23.2 (13.3)	20.7 (8.6)	0.239	0.224

Scores are presented as Mean (S.D.).

**Table 4:** Binary logistic regression predicting group allocation (MS vs. healthy controls).

Predictor	B	SE	Wald	df	*p*	OR	95% CI
9HPT non-dominant hand) (s)	0.558	0.204	7.470	1	0.006	1.747	1.171–2.607
DT walking speed (m/s)	−5.728	3.194	3.216	1	0.073	0.003	0.000–1.702
Constant	−4.838	5.499	0.774	1	0.379	–	–

Model statistics: Omnibus χ^2^(2) = 18.79, *p* < 0.001; Nagelkerke R^2^ = 0.49.

## Data Availability

Data will be made available on reasonable request from the corresponding author.
